# Zwitterionic Amino‐Acid‐Derived Polyacrylamides with a Betaine Twist – Synthesis and Characterization

**DOI:** 10.1002/marc.202400623

**Published:** 2024-09-23

**Authors:** Jonas De Breuck, Valérie Jérôme, Ruth Freitag, Meike N. Leiske

**Affiliations:** ^1^ Macromolecular Chemistry University of Bayreuth Universitätsstraße 30 95447 Bayreuth Germany; ^2^ Process Biotechnology University of Bayreuth Universitätsstraße 30 95447 Bayreuth Germany; ^3^ Bayreuth Center for Molecular Biosciences (BZMB) University of Bayreuth Universitätsstraße 30 95447 Bayreuth Germany; ^4^ Bavarian Polymer Institute Universitätsstraße 30 95447 Bayreuth Germany

**Keywords:** amino acid, betaine, chirality, post‐polymerization modification, RAFT‐polymerization, zwitterionic polymers

## Abstract

Amino‐acid‐derived polyzwitterions and polybetaines (PBs) are two promising alternatives to non‐ionic polymers, for example, to increase tumor permeability. In this study, amino‐acid‐derived polyzwitterions are synthesized and a strategy to quarternize the amine in the side chain functional group is developed to combine the advantages of both types. The functional monomer is polymerized via reversible addition−fragmentation chain‐transfer polymerization for which a kinetic study is performed. Further, the impact of the permanent positive charge on amino‐acid‐derived polyzwitterions is studied based on two zwitterionic polymers obtained via post‐polymerization modification (PPM) of Poly(*N*‐acryloxysuccinimide) to allow good comparison between methylated and non‐methylated polymers. Circular dichroism shows that the stereocenter remains intact during PPM. pH titration and ζ‐potential measurements show that the methylated polymer has a negative ζ‐potential over the measured pH range and, therefore, the polymer remains zwitterionic over a broader pH range than its non‐methylated equivalent. Both polymers are well tolerated by mammalian cells up to concentrations of 1 mg mL^−1^. The study introduces a path to a new polymer class that combines the advantages of both PBs and amino‐acid‐derived polyzwitterions and highlights the impact a permanent charge has on the physiochemical properties.

## Graphical abstract

Polyzwitterions are promising materials for applications at the bio‐interface as they can combine both – low fouling properties and target specificity. Prominent examples of polyzwitterions include non‐chiral polybetaines and chiral amino‐acid‐derived polyzwitterions. The synthesis and characterization of chiral amino‐acid‐derived polyzwitterions are reported. Chiral polybetaines are highlighted as novel materials with potential for future applications in biological environments.

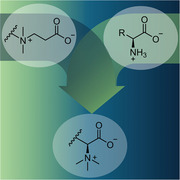

## 1 Introduction

One major challenge in nanomedicine, especially for cancer‐related treatments, is site‐specificity. Non‐ionic, biocompatible polymers having antifouling or so‐called “stealth” properties are indispensable as carriers to increase the blood retention time of disease‐treating therapeutics, that are often unstable or immiscible in the biological environment and lack tissue specificity.^[^ ^1^ ^]^ Conventional non‐ionic polymers such as poly(ethylene glycol) (PEG) are designed to prevent any interaction with biological matter (e.g., proteins or cells). However, these properties also prevent the much wanted interactions with target sites, for example, tumor cells.^[^ ^2^, ^3^ ^]^ While long‐circulating polymers such as PEG may easily access tumorous tissue through the enhanced permeation retention (EPR) effect,^[^ ^4^, ^5^ ^]^ they often lack deep penetration into the tissue.^[^ ^6^ ^]^ Polyzwitterions are one promising alternative to non‐ionic polymers for this purpose as they combine low‐fouling properties with increased tumor permeability and better uptake by cancer cells.^[^ ^7^, ^8^, ^9^, ^10^ ^]^ Like polyampholytes, polyzwitterions contain both, cationic and anionic groups along the main chain or the side chains.^[^ ^11^ ^]^ In contrast to polyampholytes, polyzwitterions are characterized by positive and negative charges at nonadjacent positions on the same pendant groups.^[^ ^12^ ^]^ Due to their ionic nature, they are often able to switch properties with changing environments.^[^ ^11^ ^]^ The different possible ionic functionalities of polyzwitterions lead to structural versatility.

Polybetaines (PBs) are one heavily researched category of polyzwitterions. They can be subdivided into three main categories: (i) poly(carboxybetaine)s (PCBs), (ii) poly(sulfobetaine)s (PSBs), and (iii) poly(phosphobetaine)s (PPBs).

PBs can be polymerized from their corresponding zwitterionic monomers using various polymerization techniques such as step‐growth polymerization, anionic polymerizations,^[^ ^11^, ^13^ ^]^ and radical polymerization.^[^ ^14^, ^15^, ^16^ ^]^ Limitations of this approach refer to the limited solubility of monomers^[^ ^17^ ^]^ or incompatibility of the ionic functionality with the opted polymerization technique. The synthesis of neutral or protected polymeric precursors and subsequent deprotection and/or post‐polymerization modification (PPM) has revealed itself useful to circumvent these issues.^[^ ^18^, ^19^, ^20^, ^21^ ^]^ One prominent example in the area of radical polymerization techniques is the PPM of poly(2‐(dimethylamino)ethyl methacrylate) (PDMAEMA) via ring opening of sultones, 2*‐oxo‐*dioxaphopholanes, and strained lactones to form the corresponding PSB, PPB, and PCB.^[^ ^11^ ^]^ From these, PCBs have shown to be highly resistant to non‐specific protein adsorption or cell adhesion.^[^ ^22^ ^]^ This makes the material promising for applications in medical diagnostics, biomaterials/tissue engineering, and drug delivery.^[^ ^23^ ^]^ Previous studies have shown that all PBs can penetrate deeply into tumor tissue, with particularly PCBs showing efficient cellular uptake into cancer cells.^[^ ^6^ ^]^

Recently, amino‐acid‐derived polyzwitterions were synthesized from different amino acids such as serine, ^[^ ^24^, ^25^, ^26^ ^]^ lysine^[^ ^24^, ^27^ ^]^ glutamine,^[^ ^28^, ^29^ ^]^ and glutamic acid.^[^ ^9^, ^10^, ^30^ ^]^ Unlike PBs, they feature an amino‐acid‐derived stereocenter, however, the primary amine functionality renders them highly responsive to changes in the pH value, which results in characterization difficulties and complicates the interpretation of structure‐property‐relationships even further. Inspired by the advantages that both classes, PBs, and amino‐acid‐derived polyzwitterions, offer, we were keen to develop an approach for the synthesis of chiral, amino‐acid‐derived PBs. Chirality plays a crucial role in further applications, particularly in the development of metal ion absorbents,^[^ ^31^ ^]^ drug delivery systems,^[^ ^32^, ^33^ ^]^ and biocompatible materials.^[^ ^34^ ^]^ This approach will create a new class of non‐biological molecules with biomimetic structures and properties. A straightforward and scalable synthesis was a specific goal. To the best of our knowledge, only chiral small molecule betaines have been synthesized from amino acids before,^[^ ^35^ ^]^ however, chiral PBs from amino acids have not been reported and will therefore be presented in this study for the first time.

**Scheme 1:**
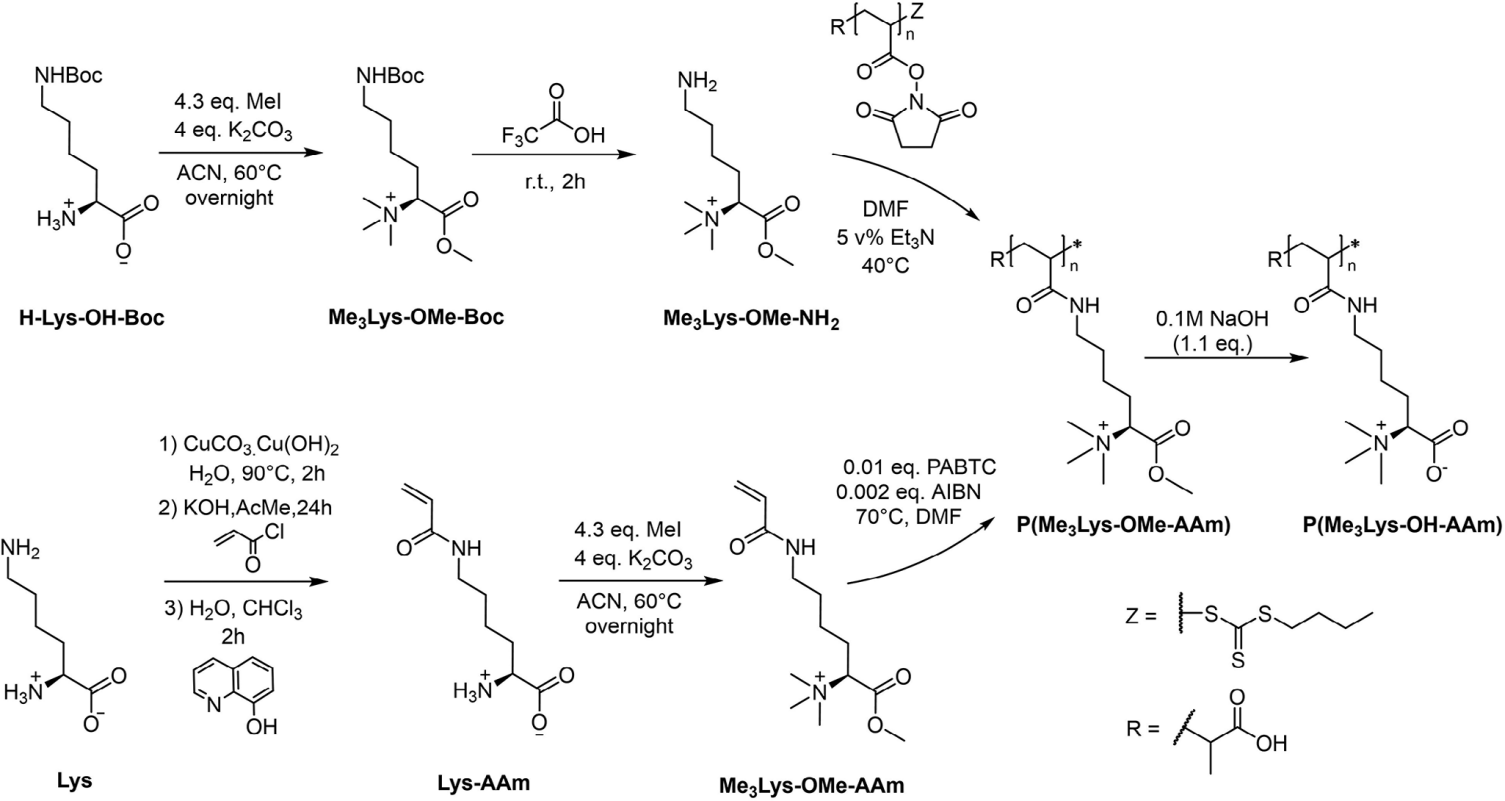
Synthesis of P(Me_3_Lys‐OH‐AAm) from monomer resembling structure and via PPM.

In our approach, it was attempted to synthesize polymers resembling amino‐acid‐derived betaine structures via two approaches: (i) polymerization of monomer resembling structures and (ii) PPM of activated polymeric precursors (**Scheme** **1**). As a result, the obtained polymers, showing stereoselectivity, underwent a preliminary biocompatibility assessment.

## 2 Results and Discussion

### 2.1 Synthesis and Characterization of Me_3_Ala‐OH as Proof‐of‐Concept Compound

**Figure 1:**
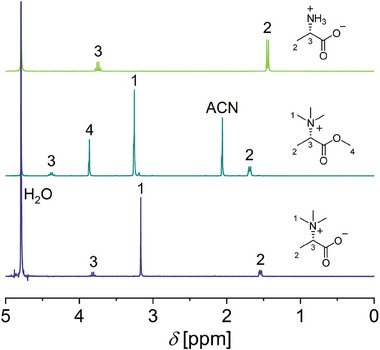
^1^H‐NMR (300 MHz, D_2_O) of Ala (top, green), Me_3_Ala‐OMe (middle, cyan), and Me_3_Ala‐OH (bottom, purple).

The polymerization of betaine‐derived monomers is often hampered by their challenging solubility behavior.^[^ ^17^ ^]^ To enable adequate solubilization of amino‐acid‐derived betaines in organic solvents, which is advantageous for controlled radical polymerization techniques as well as the PPM of amine‐reactive polymeric precursors, we attempted a quantitative quarternization of the amine as well as a methylation of the carboxylic acid yielding a methoxy group. One particular challenge of this approach is the subsequent basic hydrolysis required to yield the carboxylic acid functionality in the final polymer structure as Hofmann‐elimination might occur as an unwanted side reaction.^[^ ^36^, ^37^ ^]^ In order to find a suitable procedure to quarternize the amino acid and hydrolyze a methoxy group in the vicinity of a quaternary amine, L‐alanine (Ala) was used as a model compound for screening suitable procedures. The amine and carboxylic acid group of Ala were methylated in non‐protic hydrophilic solvent (acetonitrile) using excessive methyl iodide (MeI) in the presence of potassium carbonate to obtain the methylated Ala (Me_3_Ala‐OMe), according to an established protocol for the methylation of *tert*‐butyl (2‐aminoethyl)carbamate.^[^ ^38^ ^]^ After purification, Me_3_Ala‐OMe was analyzed via Fourier‐transform infrared spectroscopy (FT‐IR), as well as proton (^1^H‐) and carbon nuclear resonance spectroscopy (^13^C‐NMR). In the ^1^H‐NMR (**Figure** **1**) new signals appeared at *δ* = 3.25 ppm attributed to the methyl groups bound to the nitrogen (H1) and *δ* = 3.86 ppm corresponding to the newly formed methyl ester group (H4). Additionally, a shift of the signal of the α‐CH‐group (H3) from *δ* = 3.7 ppm to *δ* = 4.4 ppm was observed, suggesting successful product formation. The ^13^C‐NMR (Figure S1A, Supporting Information) confirmed these results by the newly appearing signals at *δ* = 52.0 ppm (C1) and *δ* = 53.8 ppm (C5). In addition, the ^13^C‐NMR spectrum revealed a second set of peaks, which referred to a product with hydrolyzed methoxy groups or incomplete methylation of the carboxylic acid, respectively. While in ^1^H‐NMR the presence of the hydrolyzed product was not obvious, ^13^C‐NMR measurements of the final product Me_3_Ala‐OH showed the same distinct chemical shifts. FT‐IR measurements (Figure S2, Supporting Information) further revealed a shift of the characteristic band of the C═O stretch at *ṽ =* 1747 cm^−1^, corresponding to the formation of an ester group as well as the disappearance of the NH_3_^+^‐stretch at *ṽ =* 2710 to 3100 cm^−1^.

Next, Me_3_Ala‐OMe was used to establish suitable conditions to hydrolyze the ester group, while avoiding the Hoffmann‐elimination of the quaternary amine, to obtain Me_3_Ala‐OH. Due to the expected sensitivity toward the base, we first attempted an acidic ester hydrolysis at room temperature and elevated temperatures as these were previously applied for the deprotection of dehydroalanine‐derived polymers.^[^ ^39^ ^]^ Unfortunately, the maximum degree of deprotection was 92% conversion (data not shown). For this reason, it was decided to use mild basic conditions at room temperature, which were found to obtain full conversion and consequently complete hydrolysis in the absence of Hoffmann elimination. This is shown in the ^1^H‐NMR spectrum (Figure 1)^[^ ^35^ ^]^ and in the ^13^C‐NMR spectrum (Figure S1B, Supporting Information) where the methoxy peak at *δ* = 3.9 ppm (H4) and *δ* = 53.8 ppm (C5) respectively disappeared. Complementary, FT‐IR analysis (Figures S2 and S4, Supporting Information) confirmed the hydrolysis by a shift from a C═O stretch toward *ṽ =* 1615 cm^‐1^ corresponding to a C═O stretch of a carboxylic acid.

### 2.2 Synthesis of P(Me_3_Lys‐OMe‐AAm) via Reversible Addition‐Fragmentation Chain‐Transfer (RAFT)‐Polymerization of the Monomer Resembling Structure

The successful proof of concept prompted us to develop a synthetic route to obtain chiral PBs from L‐lysine (Lys, Scheme 1). In the first route, L‐lysinyl acrylamide (Lys‐AAm) was synthesized using a cupric complexation step, according to a procedure described by *Mandal* et al.^[^ ^27^ ^]^ While in the previous publication, Lys‐AAm was used toward *tert‐*butyloxycabonyl protection, here Lys‐AAm was methylated with MeI applying the procedure optimized for the model compound to yield a vinylic betaine‐like Lys‐AAm monomer (Me_3_Lys‐OMe‐AAm).

**Figure 2:**
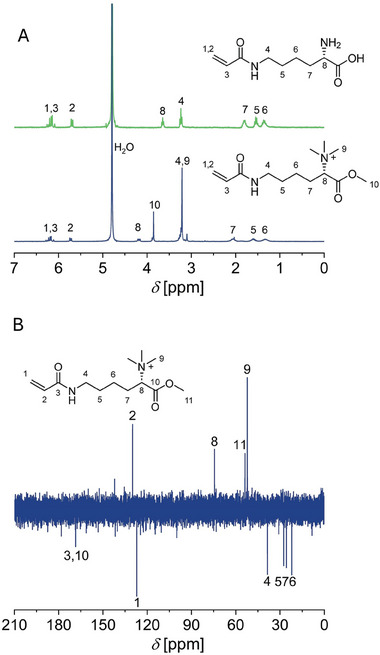
A) ^1^H‐NMR (300 MHz) of Lys‐AAm (top, green, D_2_O) and Me_3_Lys‐OMe‐AAm (bottom, blue, D_2_O) and B) ^13^C‐NMR (75 MHz) of Me_3_Lys‐OMe‐AAm (D_2_O).

In alignment with the synthesis of Me_3_Ala‐OMe, this product caused the appearance of the same new signals in the ^1^H‐NMR spectrum (**Figure** **2**A) at *δ* = 3.25 and *δ* = 3.86 ppm (H9 and H10). A shift from *δ* = 3.6 ppm to *δ* = 4.2 ppm was further observed for the α‐CH‐group (H8). The ^13^C‐NMR (Figure 2B) confirmed the formation of Me_3_Lys‐OMe‐AAm by the appearance of two signals attributed to the methyl groups bound to the quartered amine (*δ* = 52 ppm, C9) and the methyl ester (*δ* = 54 ppm, C11).

FT‐IR measurements then corroborated the formation of Me_3_Lys‐OMe‐AAm (Figures S5 and S6, Supporting Information) as the characteristic C═O and C─O absorption bands for an ester functional group appeared at *ṽ =* 1743, 1250 and 1167 cm^−1^ and a new C─N stretch absorption band corresponding to the quaternary amine appeared at *ṽ =* 1200 cm^−1^ compared to the FT‐IR spectrum of Lys‐AAm. In addition, matrix‐assisted laser‐desorption/ionization time of flight mass spectrometry (MALDI‐ToF MS, Figure S7, Supporting Information) showed a peak with the highest intensity at the expected *m/z* of 257 which corresponds with the theoretical molar mass of the monomer.

Me_3_Lys‐OMe‐AAm was subsequently polymerized in the presence of 2‐[[(butylsulfanyl)‐carbonothioyl]sulfanyl] propanoic acid (PABTC) as chain‐transfer agent (CTA) to obtain P(Me_3_Lys‐OMe‐AAm). To gain information about the polymerizability of this monomer, a kinetic study was performed and analyzed via ^1^H‐NMR (Figure S8, Supporting Information). The kinetic plots of this polymerization follow a typical trend that is generally observed in RAFT‐polymerization, where a plateau is reached after 8 h at a conversion of 87.5%.^[^ ^40^ ^]^ The polymerization also revealed a linear increase in molar mass with conversion and a narrow dispersity (Figure S9, Supporting Information).

### 2.3 Synthesis of P(Me_3_Lys‐OMe‐AAm) via Post‐Polymerization Modification (PPM)

**Figure 3:**
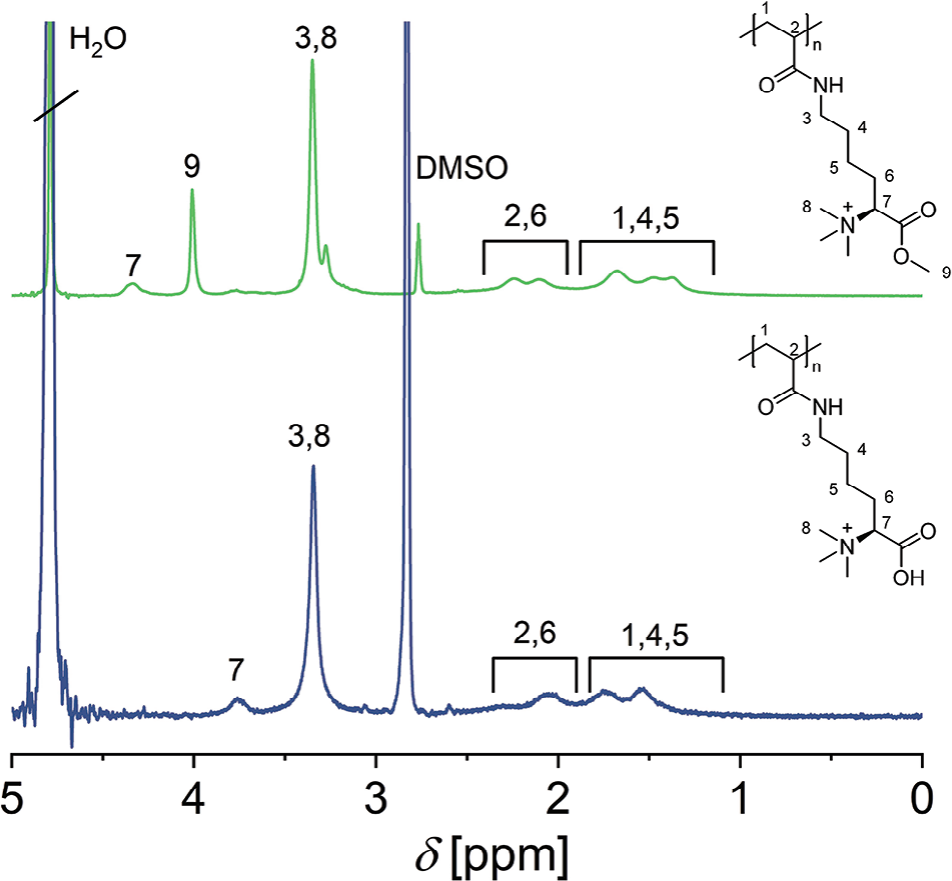
^1^H NMR (300 MHz) of P(Me_3_Lys‐OMe‐AAm) (top, green, 1:1 v [D_2_O/DMSO‐d6]) and P(Me_3_Lys‐OH‐AAm) (bottom, blue, 1:1 v [D_2_O/DMSO‐d6]).

While direct polymerization of monomer‐resembling structures is often the method of choice, the preparation of functional polymers via PPM also offers advantages. These particularly address the need for comparable polymer structures with variations in the side chain, fluorescent labeling, or copolymerization with monomers of opposing solubility. For this reason, we also investigated the potential to prepare P(Me_3_‐Lys‐OMe‐AAm) via PPM (Scheme 1). In the first step, Me_3_Lys‐OMe‐NH_2_ was synthesized by methylation of commercially available H‐Lys‐OH‐Boc using the procedure described above. ^1^H‐NMR (Figure S10, Supporting Information) confirmed the formation of a methyl ester by the appearance of a peak at *δ* = 3.9 ppm (H7) and the formation of a quaternary amine at *δ* = 3.2 ppm (H6). Subsequent acidic deprotection of the *tert*‐butyloxycarbonyl (Boc) group yielded Me_3_Lys‐OMe‐NH_2_. Successful deprotection was shown by the disappearance of the Boc peak at *δ* = 1.4 ppm (H1) and at *δ* = 27 ppm (C1) in the ^1^H‐ and ^13^C‐NMR spectrum, respectively (Figures S10 and S11, Supporting Information). P(Me_3_Lys‐OMe‐AAm) was obtained by PPM of poly(*N*‐acryloxysuccinimide) (PNAS) as a reactive precursor. PNAS was post‐modified using the synthesized Me_3_Lys‐OMe‐NH_2_ to retrieve P(Me_3_Lys‐OMe‐AAm) (Scheme 1). ^1^H‐NMR (**Figure** **3**) showed a peak shift from *δ* = 3.0 ppm to *δ* = 3.3 ppm, indicating a change in environment for the CH_2_ adjacent to the ε‐amine (H3), now positioned adjacent to the amide of P(Me_3_Lys‐OMe‐AAm). Additionally, the characteristic peak of the NAS at *δ* = 2.8 ppm^[^ ^41^ ^]^ disappeared after the reaction, indicating full conversion. According to the synthesis of Me_3_Ala‐OMe, in Figure 3 some hydrolysis could already be observed before the deprotection step. In particular, the peak at δ = 3.8 ppm could be assigned to the hydrolyzed product. P(Me_3_Lys‐OMe‐AAm) possessed a monomodal molar mass distribution determined by aqueous size‐exclusion chromatography (SEC, Figure S12, Supporting Information) indicating the absence of unwanted chain coupling during the reaction. The theoretical molar mass M_n,theo_ was estimated from the conversion of NAS during the RAFT‐polymerization (Figure S13, Supporting Information) and determined to be 49.1 kDa.

### 2.4 Effect of Methylation on the Characteristics of Chiral, Amino‐Acid‐Derived Polyzwitterions

As a second part of this study, we investigated the impact of the permanent positive charge on the physico‐chemical properties of amino‐acid‐derived polyzwitterions. For comparison, P(Boc‐Lys‐OH‐AAm) was synthesized by PPM of PNAS with commercially available Boc‐Lysine‐OH (Boc‐Lys‐OH). Similarly to the PPM of PNAS with Me_3_Lys‐OMe‐NH_2_, this reaction yielded polymers with monomodal molar mass distribution according to aqueous SEC measurements (Figure S14, Supporting Information). The theoretical molar mass M_n,theo_ was estimated from the conversion of NAS during the RAFT‐polymerization (Figure S15, Supporting Information) and determined to be 55.1 kDa. From these two modified polymers, P(Lys‐OH‐AAm) and P(Me_3_Lys‐OH‐AAm) were obtained by acidic deprotection of P(Boc‐Lys‐AAm) and by hydrolysis of P(Me_3_Lys‐OMe‐AAm) under mildly basic conditions, respectively. Successful deprotection of P(Boc‐Lys‐AAm) was verified via ^1^H‐NMR as the Boc group signal at *δ* = 1.4 ppm disappeared (H8, Figure S16, Supporting Information). Hydrolysis of the methyl ester of P(Me_3_Lys‐OMe‐AAm) was confirmed by ^1^H‐:NMR by the disappearance of the methoxy peak at *δ* = 4.0 ppm (H9, Figure 3). Furthermore, a shift from *δ* = 4.3 ppm to *δ* = 3.7 ppm was observed for the α‐CH‐group (H7).

The PPM approach allowed for a good comparison between the methylated and non‐methylated polymers. The characteristics obtained by SEC, high‐performance liquid chromatography (HPLC) as well as ζ‐potential and pH titration of the polymers of the polymers are summarized in **Table** **1**.

**Table 1:** Properties of P(Lys‐OH‐AAm) and P(Me_3_Lys‐OH‐AAm).

Polymer name	M^a)^ [Da]	DP_theo_ ^b)^	M_n,theo_ ^b)^ [kDa]	pK_a_ ^d)^	pI^e)^ [pH]	Ret. time^f)^ [min.]
P(Lys‐OH‐AAm)	200.24	193	38.9 (55.1)^c)^	2.4 (COOH) 5.8 (NH_2_)	4.3	13.1
P(Me_3_Lys‐OH‐AAm)	243.33	188	46.5 (49.1)^c)^	2.8 (COOH)	<3.0	14.1

^a)^ Molar mass of repeating unit (RU) after deprotection;

^b)^ calculated from ^1^H‐NMR;

^c)^ M_n,theo_ of modified polymer before deprotection;

^d)^ Titration of a 5 mg mL^−1^ polymer solution with 0.1 M NaOH;

^e)^ ELS measurements in water (2.0 mg mL^−1^) at varying pH. See Figure S20 (Supporting Information) for values;

^f)^ HPLC using a reversed phase column with a water/acetonitrile mixture + 0.1 V% trifluoroacetic acid (TFA) as eluent.

Since most naturally occurring proteins are optically active and show characteristic functionalities in living systems, our study aimed to obtain chiral PBs from amino acids. Herein, circular dichroism (CD) spectroscopy can verify chirality by specific conformation of the polymers in solution. Reliable assignment of the CD signals of chiral synthetic polymer seems to be difficult because unlike natural proteins most synthetic chiral polymers have a disordered conformation in solution even if the polymers possess highly stereoregular structures in the main chain.^[^ ^42^ ^]^ Consequently, CD measurements are not used to elucidate the polymeric conformation out of the CD signals in this study as the required comparison of spectra of known structures is not possible, but rather to confirm that chirality is maintained in the polymer. The CD spectrum of both polymers, P(BocLys‐OH‐AAm) and P(Me_3_Lys‐OMe‐AAm), obtained by RAFT‐polymerization of its monomer resembling structure was measured in aqueous solution (pH 6, 0.5 wt%) (Figure S17, Supporting Information). Similar CD spectrums are obtained for the deprotected polymers, P(Lys‐OH‐AAm) and P(Me_3_Lys‐OH‐AAm), obtained by PPM measured in aqueous solution (pH 7, 0.5 wt%) as better spectra were obtained at neutral pH after their deprotection (Figure S18, Supporting Information). The fact that all spectra contained peaks indicated that both the methylated and non‐methylated polymer has an overall one‐handed helical preference and, consequently, chiral repeating units, independent from their preparation method. The spectra of the polymers with a similar structure, but different synthetic approach, showed the same trend. Consequently, their secondary structures do not depend on its synthetic approach. The spectra of P(BocLys‐OH‐AAm) are also in line with previous studies by *Mandal* and co‐workers.^[^ ^27^ ^]^

To investigate the pH‐responsive properties of the polymers in water, an acid‐base titration was conducted to determine the logaritmic acidic dissociation constant (pK_a_) values. P(Me_3_Lys‐OH‐AAm) showed a pK_a_ of 2.8, attributed to the (─COOH) functional group in its side chain (Figure S19A, Supporting Information). The titration curve of P(Lys‐OH‐AAm) revealed pK_a_ values of 2.9 and 5.8, corresponding to the (─COOH) and (─NH_2_) functional groups in the side chain, respectively (Figure S19B, Supporting Information). To investigate the effect of the pH on conductivity and self‐assembly, ζ‐potential and dynamic light scattering (DLS) as well as electrophoretic light scattering (ELS) measurements were performed at different pH values. The ζ‐potential of the P(Lys‐OH‐AAm) solution varied from −27 to 21 mV upon decreasing the pH value from 9 to 3 with the isoelectric point (pI) at pH 4.3 (Figure S20, Supporting Information), which is in line with what is expected from the pK_a_ values derived from the titration curve and other previously reported amino‐acid‐derived polyzwitterions.^[^ ^9^, ^25^ ^]^ Furthermore, the rather low pI corresponds well to the acidic microenvironment of tumorous tissue. Therefore, it is expected that P(Lys‐OH‐AAm) can be applied for cellular targeting in future biological experiments, just as previous polyzwitterions with similar pH responsiveness.^[^ ^10^, ^29^ ^]^ In contrast, measurements of P(Me_3_Lys‐OH‐AAm) show a ζ‐potential value from −23  to −2 mV upon decreasing the pH value from pH 8 to 3 indicating free carboxylic groups of the polymer are located at the surface over this pH range. The ζ‐potential decreased with increasing pH as more carboxylate groups were deprotonated. The ζ‐potential over this pH range was negative as the pK_a_ of the carboxylate group was below the measured pH range. These results are in agreement with other betaine studies where a negative ζ‐potential was obtained below the pK_a_ of the anionic group.^[^ ^43^, ^44^ ^]^ This result emphasized the possible application of P(Me_3_Lys‐OH‐AAm) as a zwitterionic‐to‐cationic charge‐shifting polymer. In the zwitterionic state, it might be resistant to non‐specific protein adsorption in blood but may also switch to a positive charge to enhance cellular internalization at target sites. This would be suited for cancer therapy as tumor cells have a lower pH compared to blood and normal tissue.^[^ ^45^ ^]^ Analysis of the DLS measurement of both polymers at different pH values showed that the changes in the diameter are negligible. Solely P(Me_3_Lys‐OH‐AAm) formed assemblies with a hydrodynamic diameter (*d*_H_) of ≈160 nm at a pH 7 (Figure S21, Supporting Information), indicating aggregation of this polymer. It has already been shown that aggregation to a larger size at acidic extracellular tumor pH (pH ≈ 6.6)^[^ ^46^ ^]^ might be beneficial for retention in tumor tissues as long as they exhibit a small hydrodynamic diameter at physiochemical conditions.^[^ ^47^ ^]^ The last is tested by protein fouling tests.

In addition to pH response, it was aimed to gain information about the effect of quarternization on the hydrophobicity of the resulting polymers. It is generally known that polyzwitterions possess their highest hydrophobicity in the zwitterionic state.^[^ ^11^ ^]^ For this reason, HPLC measurements of the Cyanine‐5 (Cy5)‐labeled polymers P(Lys‐OH‐AAm)‐Cy5 and P(Me_3_Lys‐OH‐AAm)‐Cy5 were conducted to gain information about the hydrophobicity of the polymers at relevant conditions HPLC measurements using a gradient from water (0.1 V% TFA, pH 2) to acetonitrile on a reversed phase column confirmed these results by the later retention time of P(Me_3_Lys‐OH‐AAm)‐Cy5 compared to P(Lys‐OH‐AAm)‐Cy5 (Figure S22, Supporting Information; Table 1). To confirm these results, it was attempted to perform partition coefficient (PC) measurements in water at pH 4 and 7, and in Dulbecco's PBS (DPBS) (pH 7.4) Unfortunately, the fluorescence measurements were not meaningful due to an observed increase in fluorescence intensity after sample mixing with organic solvent, as compared to before mixing (data not shown). We propose that this phenomenon was attributed to incomplete phase separation. While two very different pH values were tested, the ζ‐potential and titration results have indicated that P(Me_3_Lys‐OH‐AAm) remains zwitterionic over a very broad pH range, while P(Lys‐OH‐AAm) possesses a pI around pH 4 and is, consequently, in cationic or anionic state at most conditions. This shift toward one side of charge consequently improves the hydrophilicity of the polymer.

### 2.5 Effect of Methylation on the Cytocompatibility and Protein‐Fouling of Polyzwitterions

Different polyzwitterions have shown great promise for applications at the biointerface.^[^ ^7^, ^8^, ^22^ ^]^ For this reason, we performed preliminary experiments to investigate the impact of the permanent positive charge on cell viability and aggregation in the presence of proteins. To gain information about the aggregation properties, the interactions of P(Lys‐OH‐AAm) and P(Me_3_Lys‐OH‐AAm) were studied with two representative proteins: bovine serum albumin (BSA) and lysozyme, where BSA was chosen as a negative and lysozyme a positively charged model protein.^[^ ^48^ ^]^ The fouling was analyzed in DPBS solution via DLS (Figure S23, Supporting Information) and provided information about the formation of aggregates over time as this would induce an increase in diameter in the solution over time. It was already shown that zwitterionic polymers can reduce interaction with various proteins due to both their hydration ability and the neutrality of the zwitterionic groups in the PBS buffer solution.^[^ ^49^ ^]^ It was observed that BSA does only forms very minor aggregates in the presence of P(Lys‐OH‐AAm) or P(Me_3_Lys‐OH‐AAm) after 24 h (Figure S23A–D, Supporting Information), which is in line with previous literature reports for P(Lys‐OH‐AAm).^[^ ^27^ ^]^ However, mixtures of P(Me_3_Lys‐OH‐AAm) and lysozyme indicated the formation of large nanoparticles (*d* ≈ 30 nm and 220 nm) after 30 min, which remained with *d* ≈ 30 nm even after 24 h incubation time (Figure S23E–F, Supporting Information). This can be related toward the size of nanoparticles, P(Me_3_Lys‐OH‐AAm), forms without any protein added. In comparison, the interaction between lysozyme and P(Lys‐OH‐AAm) seems to be slightly reduced, as the formed aggregates are significantly smaller (*d* < 10 nm), however, neither polymer revealed strong protein fouling. The reduced interaction of the synthesized polyzwitterions compared to polyanions in our previous study might be attributed to the positive charges of the amino groups which can influence protein‐fouling as well as protein‐repellent properties.^[^ ^50^ ^]^ This in combination with the higher hydrophilicity of P(Lys‐OH‐AAm) compared to P(Me_3_Lys‐OH‐AAm) can contribute to smaller aggregates. Therefore, it can be assumed that free, pH‐responsive amino groups provide some benefits for polymer hydration and, consequently, the low‐fouling properties of polyzwitterions. Eventually, the cell viability of MDA‐MB‐231 breast cancer cells in the presence of both polymers, which were obtained via PPM, was studied by an 3‐(4,5‐dimethylthiazol‐2‐yl)‐2,5‐diphenyltetrazolium bromide (MTT) assay (Figure S24, Supporting Information), which showed that both P(Lys‐OH‐AAm) and P(Me_3_Lys‐OH‐AAm) were well tolerated by MDA‐MB‐231 breast cancer cells at concentrations up to 1.0 mg mL^−1^ (for 24 h). These results confirmed that the methylation of chiral, amino‐acid‐derived polymers and their subsequent change in properties does not impact the tolerance by cells, thus, rendering both materials suitable for applications in the life‐science area. Future studies will evaluate the differences in cell interactions caused by quarternization to further understand these systems in more detail.

## 3 Conclusions and Outlook

This study reports for the first time the synthesis and properties of chiral, amino‐acid‐derived betaine‐like polyzwitterions. Mildly basic conditions were found to yield complete hydrolysis of the model compound Me_3_Ala‐OMe, in the absence of Hoffmann‐elimination. By transferring these conditions, Me_3_Lys‐OMe‐AAm was successfully synthesized by methylation of Lys‐AAm and, subsequently, polymerized via RAFT to obtain P(Me_3_Lys‐OMe‐AAm). In the future, this method can be used to obtain P(Me_3_Lys‐OH‐AAm) in the most direct way. This study further highlights the potential of PPM, which yields highly comparable polymers with varying side chains. A kinetic study of polymerization was successfully performed. To investigate the impact of the permanent positive charge on amino‐acid‐derived polyzwitterions, P(Lys‐OH‐AAm) and P(Me_3_Lys‐OH‐AAm) were synthesized via PPM of an amine‐reactive polymeric precursor to allow an adequate comparison between the methylated and non‐methylated compound. CD confirmed that a stereocenter was maintained via both approaches which will allow us to study the impact of chirality in these polymers in future in vitro experiments. The combination of pH titration and ζ‐potential measurements showed that P(Lys‐OH‐AAm) possessed a pI ≈ 4.3 as compared to P(Me_3_Lys‐OH‐AAm), which is expected to be present in its zwitterionic state at all pH‐values above the pK_a_ value of its (─COOH) group. Having this zwitterionic character over a broader pH range can have advantages for cellular targetability. We found that the methylation increased the hydrophobicity and the protein fouling of P(Me_3_Lys‐OH‐AAm) compared to P(Lys‐OH‐AAm), which is useful to combine them in the future with nanoparticles for cancer chemotherapy to improve their retention time in blood and subsequent tumor accumulation. Both polymers were well tolerated by cells up to concentrations of 1 mg mL^−1^. Further studies will focus on further comparing the interactions of both polymers with cells.

## Conflict of Interest

The authors declare no conflict of interest.

## Supporting information



Supporting Information

## Data Availability

The data that support the findings of this study are available from the corresponding author upon reasonable request.
