# Supplementary material for: Zwitterionic Amino‐Acid‐Derived Polyacrylamides with a Betaine Twist – Synthesis and Characterization
Source: Macromol Rapid Commun. 2024 Sep 23;46(1):2400623. doi: 10.1002/marc.202400623 (PMC11713866; doi:10.1002/marc.202400623)
Supplement: Supplementary file 1 — Supporting Information [file MARC-46-2400623-s001.pdf]

Macromolecular Rapid Communications

Supporting Information

for *Macromol. Rapid Commun.*, DOI 10.1002/marc.202400623

Zwitterionic Amino-Acid-Derived Polyacrylamides with a Betaine Twist – Synthesis and Characterization

*Jonas De Breuck, Valérie Jérôme, Ruth Freitag and Meike N. Leiske**

Supporting information

Zwitterionic amino-acid-derived polyacrylamides with a betaine twist – Synthesis and characterization

Jonas De Breuck,[a] Valérie Jérôme,[b] Ruth Freitag,[b,c] Meike N. Leiske[a,d,]*

[a] Macromolecular Chemistry, University of Bayreuth, Universitätsstraße 30, 95447 Bayreuth, Germany

[b] Process Biotechnology, University of Bayreuth, Universitätsstraße 30, 95447 Bayreuth, Germany

[c] Bayreuth Center for Molecular Biosciences (BZMB), University of Bayreuth, Universitätsstraße 30, 95447 Bayreuth, Germany

[d] Bavarian Polymer Institute, Universitätsstraße 30, 95447 Bayreuth, Germany

*correspondence to: meike.leiske@uni-bayreuth.de

1. Experimental Part

1.1. Materials and instrumentation

1.1.1. Materials

Acetonitrile (99.9%), diethyl ether (99.5%), dimethylformamide (99.5%), methanol (99.9%), sodium hydrogen carbonate (>99.7%) and methyl iodide (99.0%) were purchased from FisherScientific. Chloroform (99.8%), dichloromethane (99.8%), isopropanol (99.9%), sodium hydroxide (99.5%), potassium hydroxide (99.5%), Hydrochloric acid (32%), Dulbecco's Modified Eagle Medium (DMEM) Low Glucose, Fetal Bovine Serum Advanced, Penicillin/Streptomycin (Pen/Strep), Dulbecco's PBS, Bovine serum albumin (BSA) and lysozyme, from which the last two obtained as lyophilized powders, were purchased from VWR Chemicals. Triethylamine (99.5%), potassium carbonate (99.0%), 8-hydroxyquinoline (for synthesis) and Anisole (99.0%) were purchased from Sigma Aldrich. Thiazolyl blue tetrazolium bromide (MTT) was purchased from TCI Chemicals. Cyanin5-amine (95.0%) was purchased from Lumiprobe. H-Lys(Boc)-OH (97.0%) and *N*-hydroxysuccinimide (98.0%) were purchased from BLD Pharmatech ltd. Trifluoroacetic acid (99.0%) was purchased from abcr. Acryloyl chloride (96%) was purchased from ThermoScientific. Cupric (II) carbonate basic was purchased from Honeywell. Boc-Lys-OH (98%) and L-lysine hydrochloride (98%) were purchased from Carbolution.

Azobis(isobutyronitril) (AIBN) (Fluka or Sigma Aldrich, 98.0%) was recrystallized from methanol before use. *N*-Acryloxysuccinimide (NAS)[37] and 2-{[(butylsulfanyl)-carbonothiosyl]sulfanyl}propanoic acid (PABTC)[44] were synthesized and characterized following known procedures in literature.

1.1.2. Proton nuclear resonance (^{1}H-NMR) and carbon nuclear resonance (^{13}C-NMR) spectroscopy

^{1}H- and ^{13}C-NMR spectra of all compounds were recorded on a Bruker Avance 300 (300 MHz) spectrometer equipped with a BACS-120 autosampler and a ^{1}H, ^{13}C, ^{19}F and ^{31}P-BBO probe at room temperature.

1.1.3. Size-exclusion chromatography (SEC)

Aqueous SEC measurements of P(Me_3Lys-OMe-AAm) and P(Me_3Lys-OMe-AAm)-Cy5 were performed on an instrument consisting of a column set with a NOVEMA Max precolumn (particle size = 10 µm) and three NOVEMA Max main columns (particle size = 10 µm, 1 × 100 Å; 2 × 3000 Å) with separation range from 100 to 3 000 000 Da (PSS, Mainz, Germany) together with a variable wavelength detector (1200 Series, Agilent Technologies). As solvent 80:20 Water/ACN mixture + 0.1 M NaCl + 0.1 V% TFA was used (for dissolving polymer and as eluting solvent) with a flow rate of 0.5 mL/min and the columns were maintained at room temperature. As internal standard ethylene glycol (HPLC grade) was used. The calibration was done with narrowly distributed Poly(2-vinylpyridine) (narrowly distributed P2VP homo-polymers, PSS calibration kit). An injection volume of 60 µL was used for the measurements. The samples were dissolved with a concentration of 2 mg mL^{-1} and filtered through a 0.22 µm PTFE Nylon filter before analysis. The UV-detector was set to λ = 600 nm for measurements of Cy5-labeled polymers.

Aqueous SEC measurements of P(BocLys-OH-AAm) and P(BocLys-OH-AAm)-Cy5 were performed on an instrument consisting of a column set with a SUPREMA precolumn (particle size = 5 µm), one SUPREMA (particle size = 5 µm, 30 Å) and two NOVEMA Max main columns (particle size = 5 µm, 2 × 1000 Å) with separation range from 100 to 1 000 000 Da (PSS, Mainz, Germany) together with a variable wavelength detector (1200 Series, Agilent Technologies). As solvent 65:35 Water/ACN mixture + 50 mM LiBr + 0.1 M $NaHCO_3$ was used (for dissolving polymer and as eluting solvent) with a flow rate of 0.8 mL min^{-1} and the columns were maintained at room temperature. As internal standard ethylene glycol (HPLC grade) was used. The calibration was done with narrowly distributed poly(ethylene glycol) (narrowly distributed PEG homo-polymers, PSS calibration kit). An injection volume of 60 µL was used for the measurements. The samples were dissolved with a concentration of 2 mg mL^{-1} and filtered through a 0.22 µm PTFE Nylon filter before analysis. The UV-detector was set to λ = 600 nm for measurements of Cy5-labeled polymers.

DMF SEC measurements of PNAS was performed on an instrument consisting of a GRAM precolumn (particle size = 10 µm) and two GRAM main columns (Particle size= 10 µm, 1 × 100 A, 1 × 3000 A) with separation range from 100 to 5 000 000 Da (PSS, Mainz, Germany) together with a diode array and refractive index detector (1260 Infinity, Agilent Technologies). As solvent DMF (HPLC grade) with lithium bromide was used (for dissolving polymer and as eluting solvent) with a flow rate of 0.5 mL/min and the columns were maintained at room temperature. As internal

standard Toluene (HPLC grade) was used. The calibration was done with narrowly distributed polystyrene homo-polymers (PSS calibration kit). An injection volume of 20 µL was used for the measurements. The samples were dissolved with a concentration of 2 mg mL^{-1} and filter through a 0.22 µm PTFE Nylon filter before analysis.

1.1.4. High-performance liquid chromatography (HPLC)

HPLC measurements were conducted with a Jupiter 5 µm C18 300 Å LC column (250 × 4.6 mm). The run time was 35 min. A mixture of 0.1 v/v% TFA in water and acetonitrile served as eluent (**Table S1**). The fluorescence detected was set to λ_{ex} = 640 nm and λ_{em} = 680 nm with 1× gain. The ELSD detector was set to λ = 215 nm.

Table S1. Composition of HPLC eluent at indicated time points.

Time [min]	Fraction of 0.1v% aqueous TFA [%]	Fraction of acetonitrile [%]
0	98.0	2.0
5	50.0	50.0
10	0.0	100.0
17	0.0	100.0
25	98.0	2.0
35	98.0	2.0

1.1.5. Dynamic light scattering (DLS)

DLS was measured on a Zetasizer Nano-ZS Malvern apparatus (Malvern Instruments Ltd) using disposable cuvettes. The excitation light source was a He–Ne laser at 633 nm and the intensity of the scattered light was measured at an angle of 173°. This method measures the rate of intensity fluctuation, and the size of the particles is determined through the Stokes–Einstein equation. The concentration of the polymer solution was 2 mg mL^{-1} in all cases.

1.1.6. Electrophoretic light scattering (ELS)

ELS was used to measure the zeta potential (ζ). The measurement was also performed on a Zetasizer Nano ZS by applying laser Doppler velocimetry. For each measurement, 20 runs were carried out using the slow-field reversal and the fast-field reversal mode at 150 V. Each experiment was performed in triplicates at 25 °C. The ζ-potential was calculated from the electrophoretic

mobility (μ) according to the Henry equation. Henry coefficient f(ka) was calculated according to Ohshima.[45] The concentration of the polymer solution was 2 mg mL^{-1} in all cases.

1.1.7. Fourier-Transform Infrared Spectroscopy (FT-IR)

IR spectra were recorded from solids on a Perkin Elmer Spectrum 100 FT-IR spectrometer in attenuated total reflection (ATR) mode.

1.1.8. Matrix-assisted laser desorption/ionization time-of-flight mass spectroscopy (MALDI-ToF MS)

measurements were performed using a Bruker AutoFlex Max mass spectrometer equipped with a Smart-beam II laser. The analyte was embedded in the matrix/salt combination of 2,3-Dihydroxybenzoic acid (DHB)/ Pottasium trifluoroacetate (KTFA) in the matrix:analyte:salt mass ratio 20:3:1.

1.2. Synthesis and characterization

1.2.1. Synthesis of PNAS

In a 2 mL reaction vessel (Biotage), 507 mg NAS (3 mmol, 200 equiv.), 0.082 mg AIBN (5×10^{-7} mol, 0.1 equiv.), 1.19 mg PABTC (5×10^{-6} mol, 1 equiv.) were dissolved in 1 mL DMF and sealed with a rubber septum. A sample was taken (t = 0 h) for ^{1}H-NMR spectroscopy in DMSO-d6 or $CDCl_3$ as indicated to determine the conversion by comparing the sample with a sample at the end of the reaction. The reaction mixture was deoxygenated with Ar for 30 min and subsequently placed in a preheated sand bath and stirred at 70 °C for 24 h. The reaction was terminated by cooling to RT and purging air in. A sample was taken (t = 24 h) and analyzed *via* ^{1}H-NMR spectroscopy in DMSO-d6. The crude polymer was precipitated in ice-cold diethyl ether twice. After centrifugation (5000 rpm, 6 min), the supernatant was discarded, and the polymer was dried under reduced pressure overnight to obtain the product as a yellow viscous oil. The key properties are summarized in Table S2.

Table S2. Properties of PNAS used for the synthesis of different amino-acid-functionalized polymers.

	^{1}H-NMR[a]			SEC[c]	
PNAS for PPM towards:	Conv. [%]	DP_{theo}[b]	$M_{n,theo}$[b] [KDa]	$M_{n,app}$ [kDa]	Đ
P(Lys-OH-AAm)	96.4	193	32.8	18.4	1.52
P(Me_3Lys-OH-AAm)	94.2	188	32.0	16.5	1.53

[a] ^{1}H NMR (300 MHz). [b]calculated from monomer conversion. [c]SEC in DMF (PS calibration).

1.2.2. Post-polymerization modification of PNAS with Boc-Lys-OH

In a 25 mL round bottom flask, PNAS (200 mg, 6.8×10^{-6} mol, 1.00 equiv.) was dissolved in anhydrous DMF (9.5 mL). 500 µL triethylamine were added. After that, Boc-Lys-OH (297 mg, 1.21 mmol, 205 equiv. per polymer) was added, the reaction vial was closed with a septum and the suspension was stirred at 40 °C for 24 h. Then, the solid was filtered off and the remaining solution was dialyzed (RC, MWCO 3.5 kDa, SpectraPor) against 1 L deionized water for three days with daily water changes. Upon lyophilization, the product was yielded as an off-white powder.

SEC (PEG-cal.): $M_{n,app}$ = 37,100 g mol^{-1}; Đ = 1.50.

1.2.3. Post-polymerization modification of PNAS with Me_3Lys-OMe-NH_2

In a 25 mL round bottom flask, PNAS (300 mg, 9.36×10^{-6} mol, 1.00 equiv.) was dissolved in anhydrous DMF (4.75 mL). 250 µL triethylamine were added. After that, Me_3Lys-OMe-NH_2 (395 mg, 1.94 mmol, 205 equiv. per polymer) was added, the reaction vial was closed with a septum and the suspension was stirred at 40 °C for 24 h. Then, the solid was filtered off and the remaining solution was dialyzed (RC, MWCO 3.5 kDa, SpectraPor) against 1 L deionized water for three days with daily water changes. Upon lyophilization, the product was yielded as a yellowish powder. The success of the reaction was verified *via* ^{1}H-NMR and FTIR.

SEC (P2VP-cal.): $M_{n,app}$ = 10,100 g mol^{-1}; Đ = 1.96.

1.2.4. *Post-polymerization modification of PNAS with Cy5-amine and BocLys-OH or Me_3Lys-OMe-NH_2*

The labeling process is exemplarily described for PNAS yielding P(Me_3Lys-OMe-AAm)-Cy5. In a small reaction vessel, 45 mg of PNAS (1.39×10^{-6} mol, 1.0 equiv.) and 1 µL of Et_3N (7.0×10^{-6} mol, 5 equiv. per polymer) were dissolved in anhydrous 1 mL DMF and stirred at RT in the dark for 24 h. Subsequently, a 10 mg mL^{-1} solution of Cy5-amine (1.0 mg, 1.7×10^{-6} mol, 1.2 equiv. per polymer) in anhydrous DMF was added. The reaction was stirred at RT in the dark overnight. Then, 62 mg of Me_3Lys-OMe-NH_2 (3.05×10^{-4}, 220 equiv. per polymer) and 55 µL of Et_3N (3.95×10^{-4}, 284 equiv. per polymer) were added to the reaction mixtures which was stirred at RT in the dark for an additional 24 h. After that, the solid was filtered off and the remaining solution was dialyzed (RC, MWCO 3.5 kDa, SpectraPor) against 1 L deionized water for four days (until water outside membrane was colourless) with daily water changes in the dark. Upon lyophilization, the product was yielded as a blue powder.
P(Me_3Lys-OMe-AAm)-Cy5: Aqueous (SEC) (P2VP-cal.): $M_{n,app}$ = 7,600 g mol^{-1}; Đ = 2.53
P(BocLys-OH-AAm)-Cy5: Aqueous (SEC) (PEG-cal.): $M_{n,app}$ = 3,700 g mol^{-1}; Đ = 1.32

1.2.5. *Acidic deprotection of P(Boc-Lys-AAm) yielding P(Lys-OH-AAm)*

In a reaction vessel, 250 mg of polymer were dissolved in 1 mL trifluoroacetic acid, and the reaction was stirred at room temperature for 2 h. Dye-labelled polymers were stirred in the dark in a 50/50 V% [TFA:water] mixture. Subsequently, the reaction mixture was diluted with 1 mL EtOH and the polymer was precipitated in 40 mL ice-cold diethyl ether. Then, the suspension was centrifuged (5000 rpm, 6 min) and the supernatant was discarded. The polymer was re-dissolved in H_2O and freeze-dried to obtain the product as a white powder.
The success of the deprotection of the polymer was analyzed by ^{1}H-NMR in D_2O, showing the disappearance of the BOC, signal at $\delta = 1.4$ ppm.

1.2.6. *Basic deprotection of P(Me_3Lys-OMe-AAm) yielding P(Me_3Lys-OH-AAm)*

In a reaction vessel, 100 mg of P(Me_3Lys-OMe-AAm) (2.15×10^{-6} mol, 1.0 equiv.) were dissolved in 5 mL of 0.1 N NaOH solution (5×10^{-4} mol, 230 equiv. per polymer). The reaction mixture was stirred at room temperature for 24 h. Dye-labeled polymers were stirred in the dark. Subsequently, the solution was dialyzed (RC, MWCO 3.5 kDa, SpectraPor) against 1 L deionized water for four days with daily water exchanges (Cy5-labeled polymers were dialyzed in the dark). Upon

lyophilization, the product was recovered as a white to yellowish powder (blue powder for the Cy5-labeled polymers).

1.2.7. pH response

The response of the different polymers to the pH value was investigated by titration. A 5 mg mL^{-1} solution of the polymer in deionized water was prepared and 20 µL 1.0 M aq. HCl were added to obtain a starting pH value < 3. Subsequently, the solution was titrated with 0.1 M aq. NaOH until a pH value > 9 was reached. Measurements were done with a Mettler Toledo digital pH meter.

1.3. Biological testing

1.3.1. Protein fouling

Fouling of P(Me_3Lys-OH-AAm) and P(Lys-OH-AAm) by proteins (BSA and lysozyme) was analyzed by DLS measurements.

Stock solutions of the polymers and the proteins were prepared separately in DPBS (1 mg mL^{-1}) and mixed in a ratio of 1:1, resulting in final concentrations of c = 0.5 mg mL^{-1}. The samples were analyzed immediately after mixing (t = 0 h) and at indicated time points (0.5 h, 1 h, 2 h, 4 h, 24 h) thereafter. In between measurements the samples were incubated at 37 °C whilst being shaken at 100 rpm. Measurements were conducted at 37 °C with five measurements and three runs each. After the measurement, samples were placed back into the incubator immediately.

1.3.2. Cell culture

MDA-MB-231 cells (HTB-26, ATCC) were maintained in DMEM cell culture medium supplemented with 10% fetal calf serum (FCS), 100 mg mL^{-1} streptomycin, 100 U mL^{-1} penicillin, and 4 mM glutamine. Cells were cultivated at 37 °C in a humidified 5% CO_2 atmosphere.

1.3.3. Cell viability

The cytotoxicity of P(Me_3Lys-OH-AAm) and P(Lys-OH-AAm)), which were obtained *via* PPM approach, were tested using MDA-MB-231 breast cancer cells. The polymers stock solutions were prepared at concentrations ≤ 20 mg mL^{-1}. The polymers were tested in a concentration range from 0 to 1.0 mg mL^{-1}. The cells (10^4 cells per well) were seeded in culture medium (100 µL per well)

in 96-well plates and allowed to adhere overnight. No cells were seeded in the outer wells, these were just filled with water. The medium was subsequently removed and replaced by fresh polymer-containing media. Then, the cells were incubated for an additional 24 h. After that, the medium was removed, the cells were washed with 100 µL Dulbecco's PBS (DPBS) and fresh culture medium containing thiazolyl blue tetrazolium bromide (MTT) (concentration: 1 mg mL^{-1}) was added (100 µL per well). Note: MTT (50 mg) was dissolved in 10 mL of sterile DPBS and 1 to 5 diluted in culture medium prior to use in this assay. After incubation for 3 h at 37 °C, the medium was removed and 100 µL of isopropanol (^{i}PrOH) was added to each well. Then, the plates were gently shaken in the dark for 15 minutes to dissolve the formazan crystals. Quantification was done by measuring the absorbance at λ = 580 nm using a microplate reader (Genios Pro, Tecan). Untreated cells on the same plate served as negative control (100% viability), cells treated with 20% DMSO as positive control (0% viability), and wells without cells as background. For data evaluation, Excel was used. For each dilution step, 6 wells were measured and performed in triplicate. The cell viability was calculated according to equation (2).

$$\%\, Cell\ viability = \frac{(Abs.sample - Abs.background)}{(Abs.negative\ control - Abs.background)} \times 100 \qquad (2)$$

2. Results

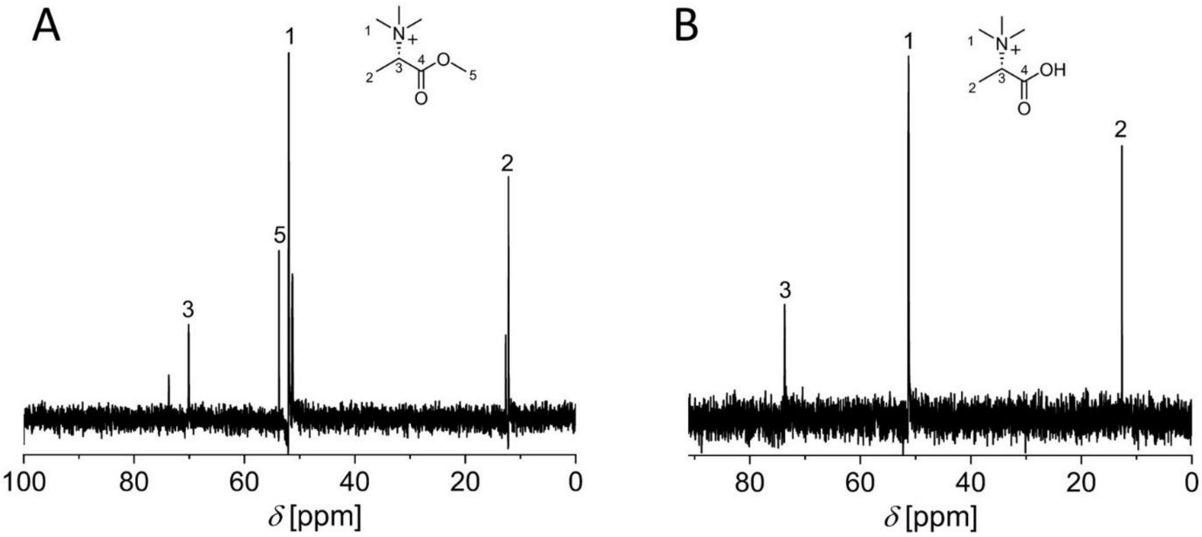

Figure S1. ^{13}C-NMR (75 MHz, D_2O) of A: Me_3Ala-OMe and B: Me_3Ala-OH.

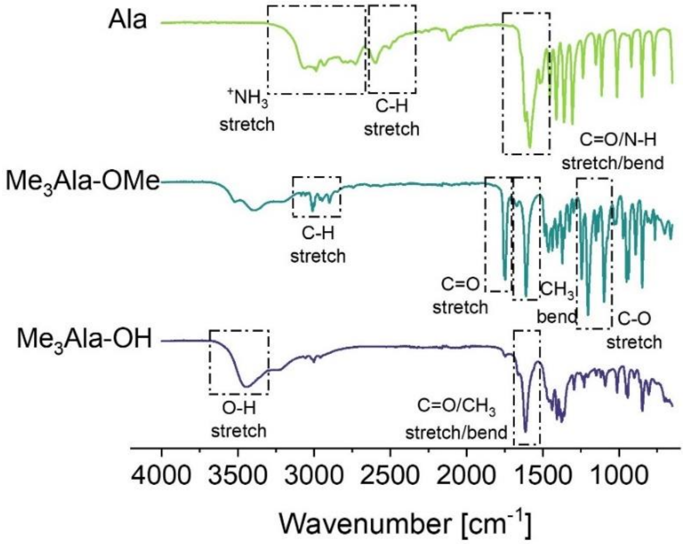

Figure S2. FT-IR spectra of Ala (top, green), Me_3Ala-OMe (middle, cyan) and Me_3Ala-OH (bottom, purple).

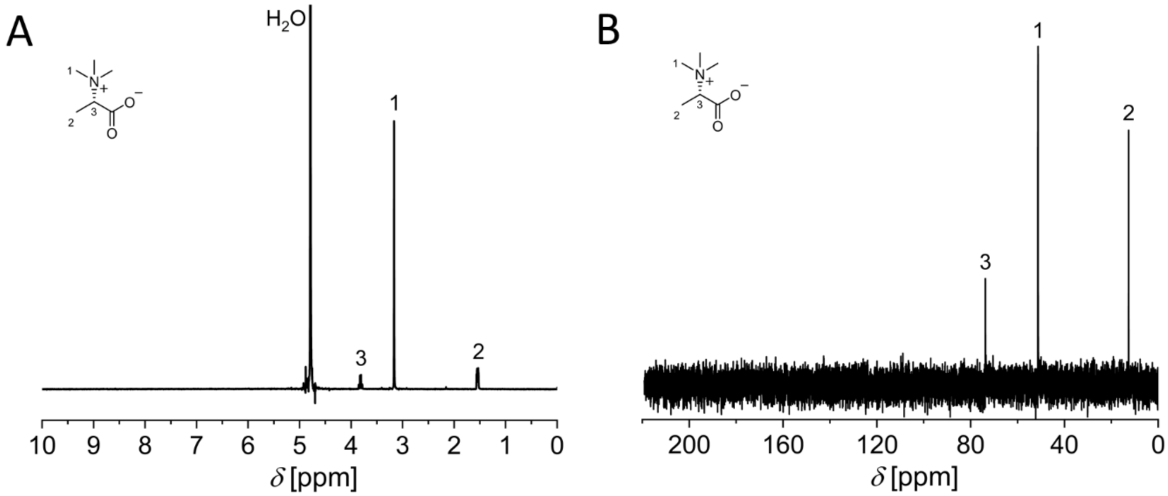

Figure S3. *A: ^{1}H-NMR (300 MHz, D_2O) Me_3Ala-OH and B: ^{13}C-NMR (75 MHz, D_2O).*

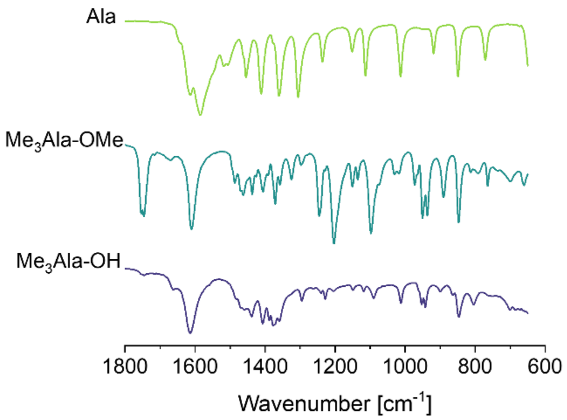

Figure S4. 1800-600 cm^{-1} region of FT-IR spectra of Ala (top, green), Me_3Ala-OMe (middle, cyan) and Me_3Ala-OH (bottom, purple).

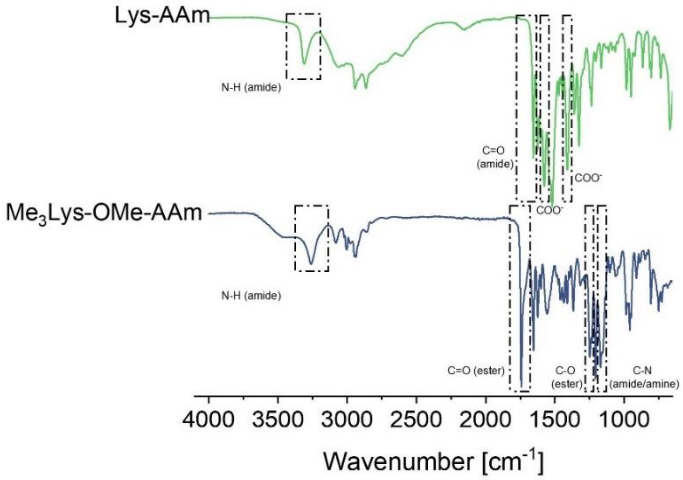

Figure S5. FT-IR spectra of Lys-AAm (top, green) and Me_3Lys-OMe-AAm (bottom, blue).

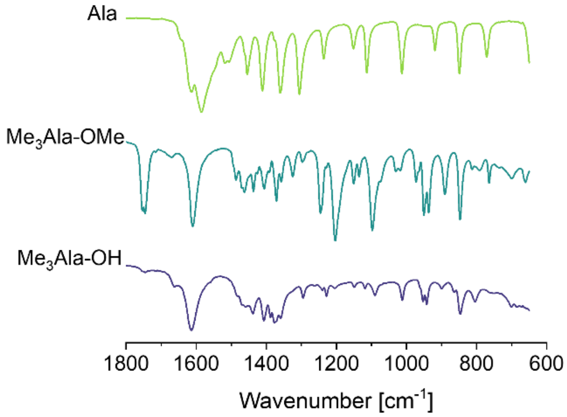

Figure S6. 1800-600 cm^{-1} region of Lys-AAm (top, green) and Me_3Lys-OMe-AAm (bottom, blue).

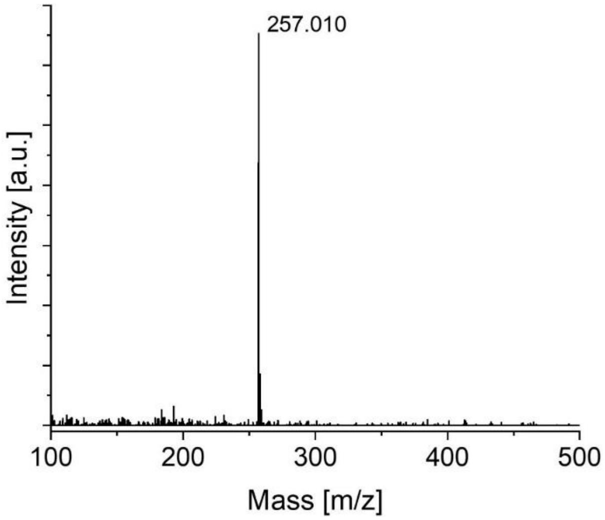

Figure S7. MALDI-ToF mass spectrum of Me_3Lys-OMe-AAm.

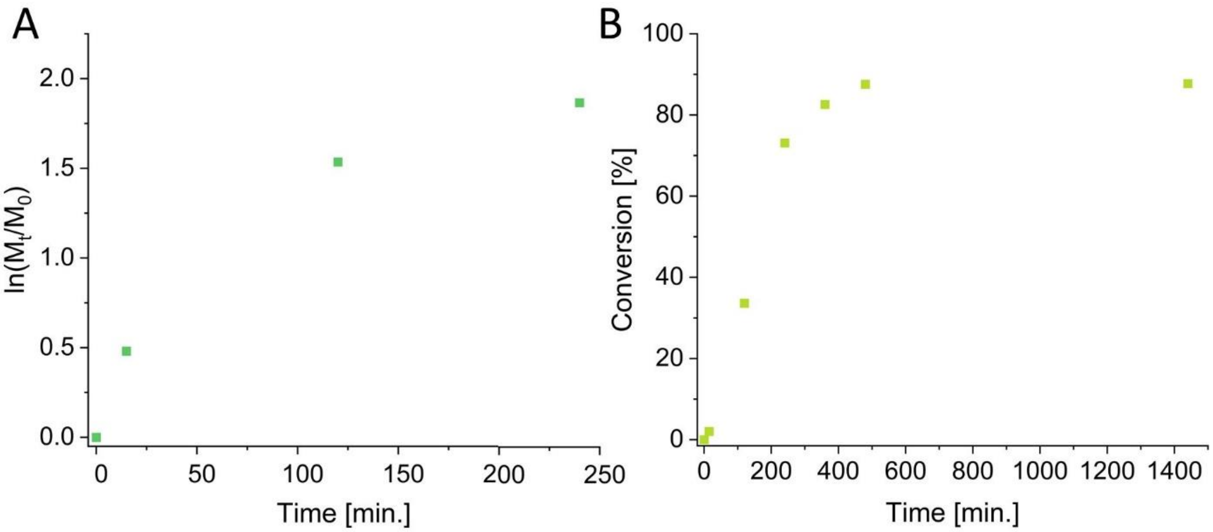

Figure S8. Time-dependent polymerization kinetics on the RAFT-polymerization of Me_3Lys-OMe-AAm using PABTC as CTA ([M]/[CTA]/[I] = 100:1:0.2). Polymerizations were conducted in DMF at 70°C using AIBN as initiator. The monomer conversion was calculated by ^{1}H-NMR, using trioxane as an internal standard. A: logarithmic plot of the polymerization kinetics until 240 min. (linear region). B: monomer conversion over time.

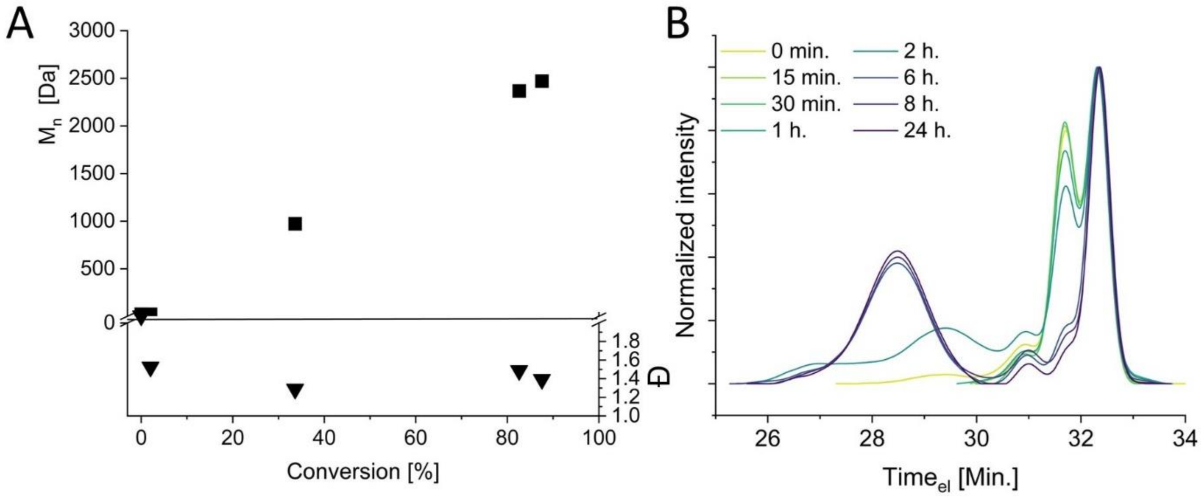

Figure S9. A: development of apparent molecular weight and polydispersity index (PDI) of P(Me_3Lys-OMe-AAm) on conversion B: Aqueous SEC traces of kinetic studies of Me_3Lys-OMe-AAm in 80:20 V% [H_2O/ACN] + 0.1M NaCl + 0.3v% TFA (standard: P2VP).

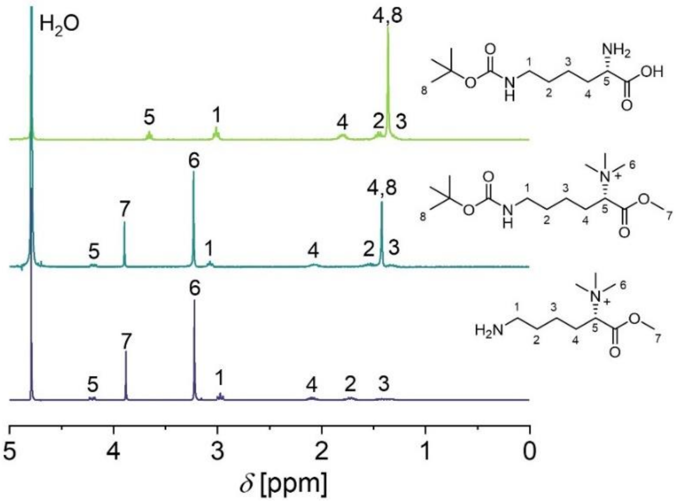

Figure S10. ^{1}H-NMR (300 MHz, D_2O) of H-Lys-OH-Boc (top, green), Me_3Lys-OMe-Boc (middle, cyan) and Me_3Lys-OMe-NH_2 (bottom, purple).

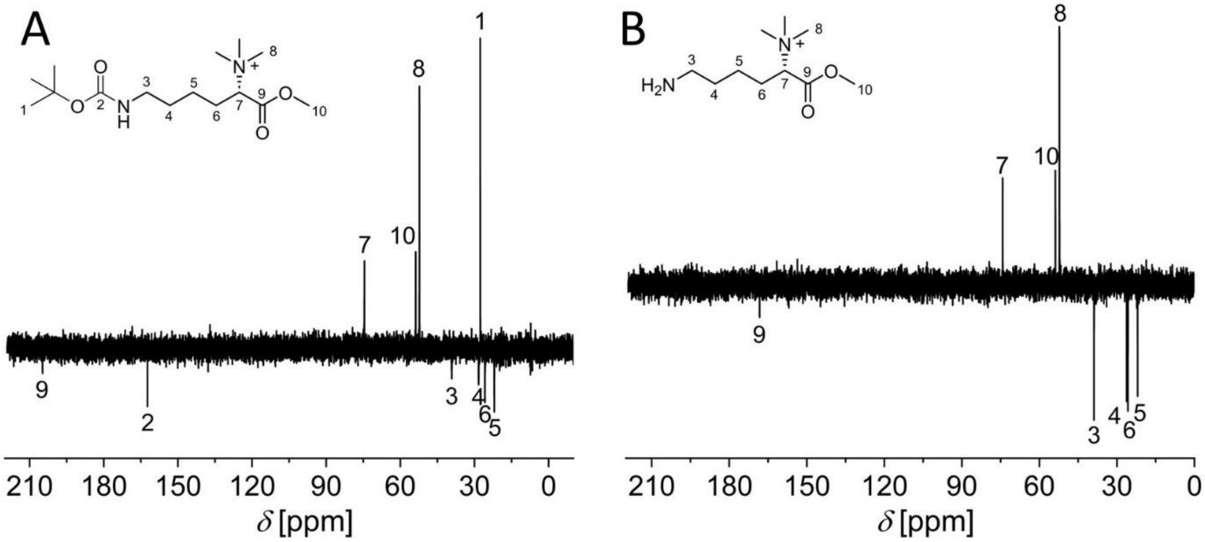

Figure S11. ^{13}C-NMR (75 MHz, D_2O) of A: Me_3Lys-OMe-Boc and B: Me_3Lys-OMe-NH_2.

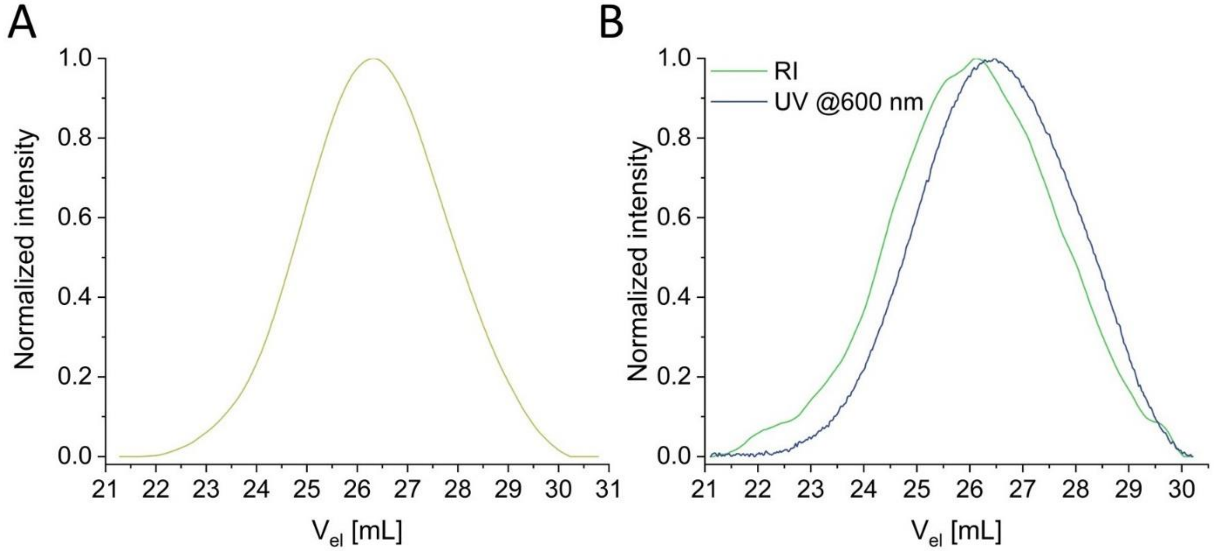

Figure S12. Aqueous SEC elugram of A: P(Me_3Lys-OMe-AAm) and B: P(Me_3Lys-OMe-AAm)-Cy5 in 80:20 V% [H_2O/ACN] + 0.1M NaCl + 0.3v% TFA (standard: P2VP).

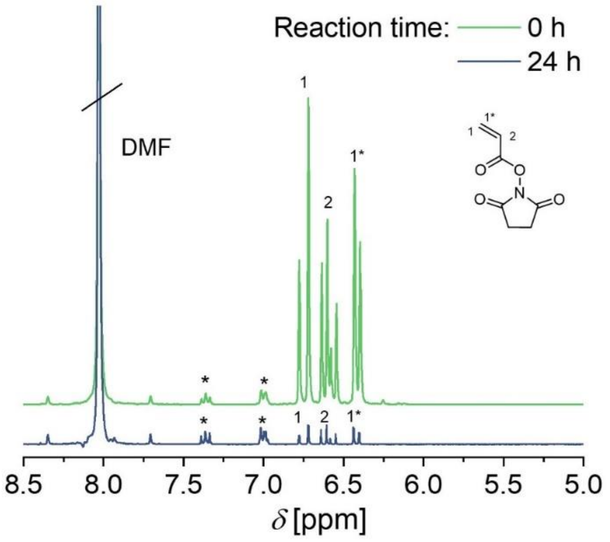

Figure S13. ^{1}H-NMR (300 MHz, DMSO-d6) of reaction mixtures during RAFT synthesis of PNAS at t = 0 h (top, green) and t = 24 h (bottom, blue) using anisole as internal standard to follow conversion (indicated by *). PNAS was used in PPM to obtain P(Me_3Lys-OMe-AAm).

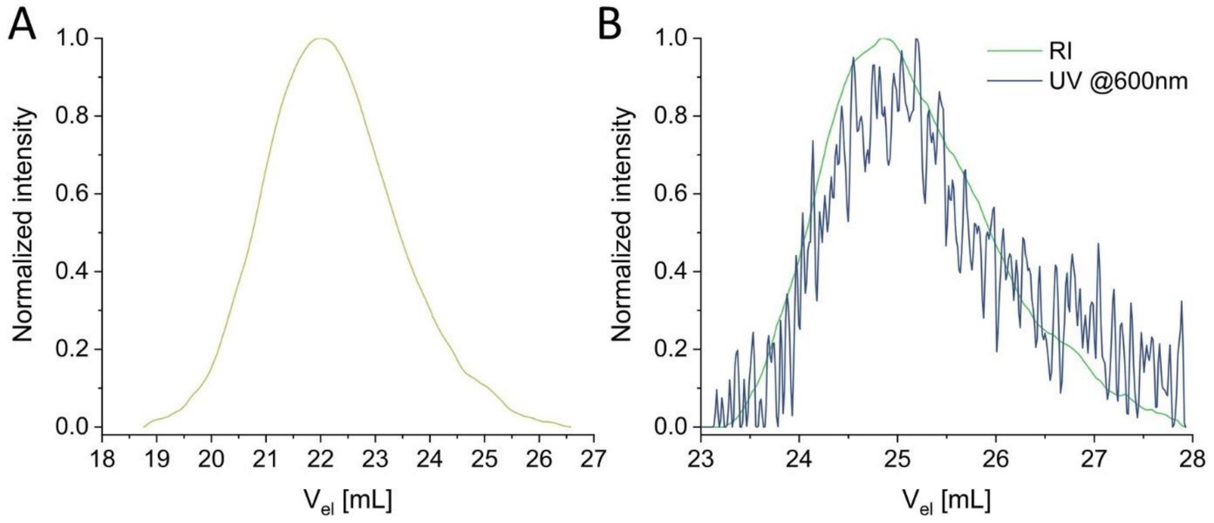

Figure S14. Aqueous SEC elugram of A: P(BocLys-OH-AAm) and B: P(BocLys-OH-AAm)-Cy5 in 65:35 [H_2O/ACN] mixture + 50 mM LiBr + 0.1 M $NaHCO_3$ (Standard: PEG).

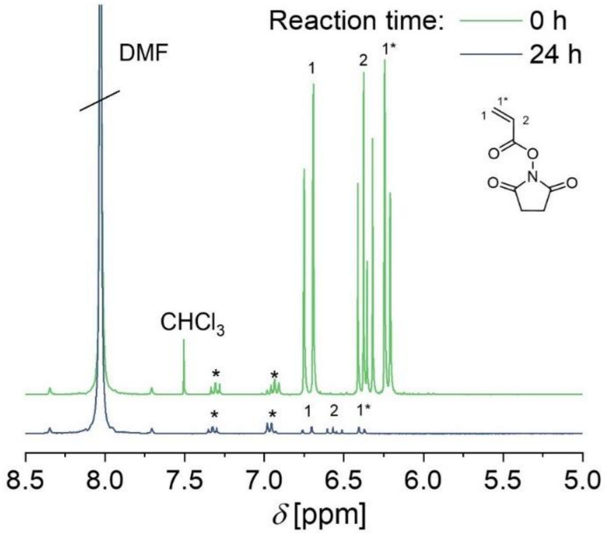

Figure S15. ^{1}H-NMR (300 MHz) of reaction mixtures during RAFT synthesis of PNAS at t = 0 h (top, green, $CDCl_3$) and t = 24 h (bottom, blue, DMSO-d6) using anisole as internal standard to follow conversion (indicated by *). PNAS was used in PPM to obtain P(Boc-Lys-OH-AAm).

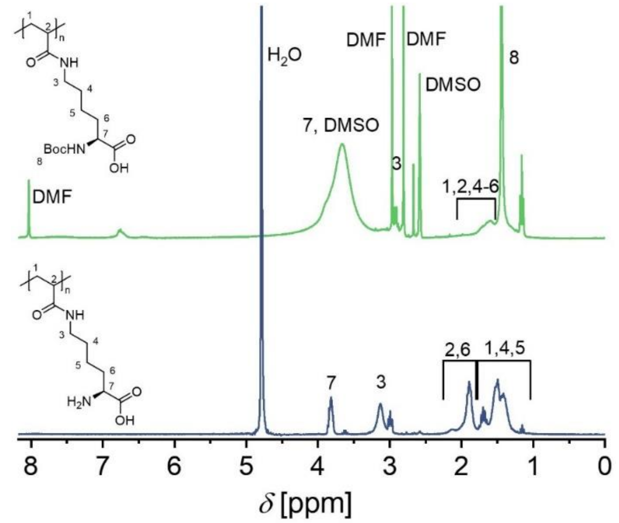

Figure S16. ^{1}H-NMR (300 MHz, D_2O) of P(Boc-Lys-OH-AAm) (top, green) and P(Lys-OH-AAm) (bottom, blue).

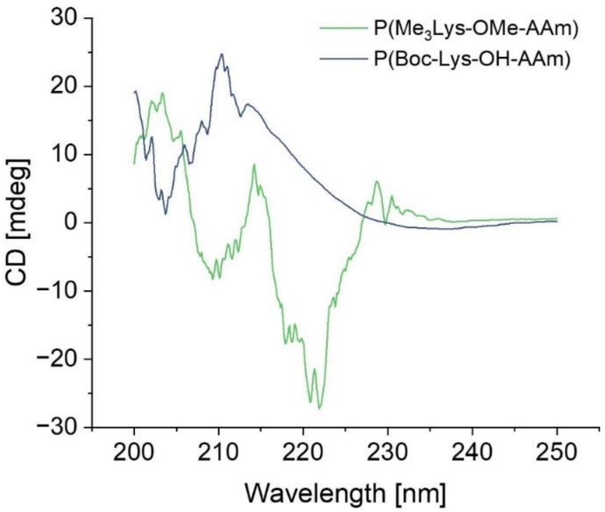

Figure S17. CD spectrum of P(Boc-Lys-OH-AAm) (blue) and P(Me_3Lys-OMe-AAm) (green), obtained by RAFT of monomer resembling structure, in water at pH 6.

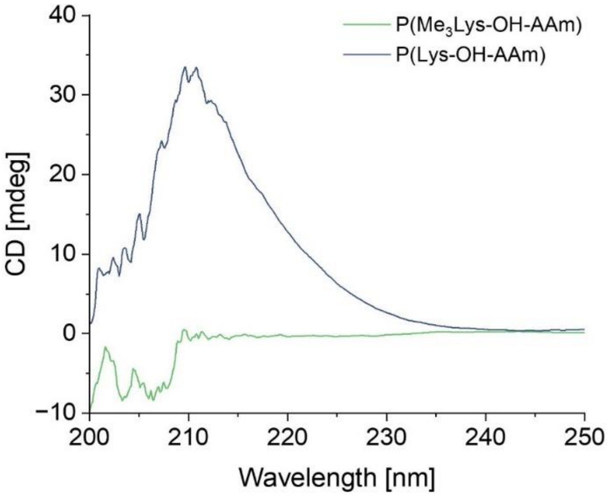

Figure S18. CD spectrum of P(Lys-OH-AAm) (blue) and P(Me_3Lys-OH-AAm) (green), obtained by PPM approach, in water at pH 7.

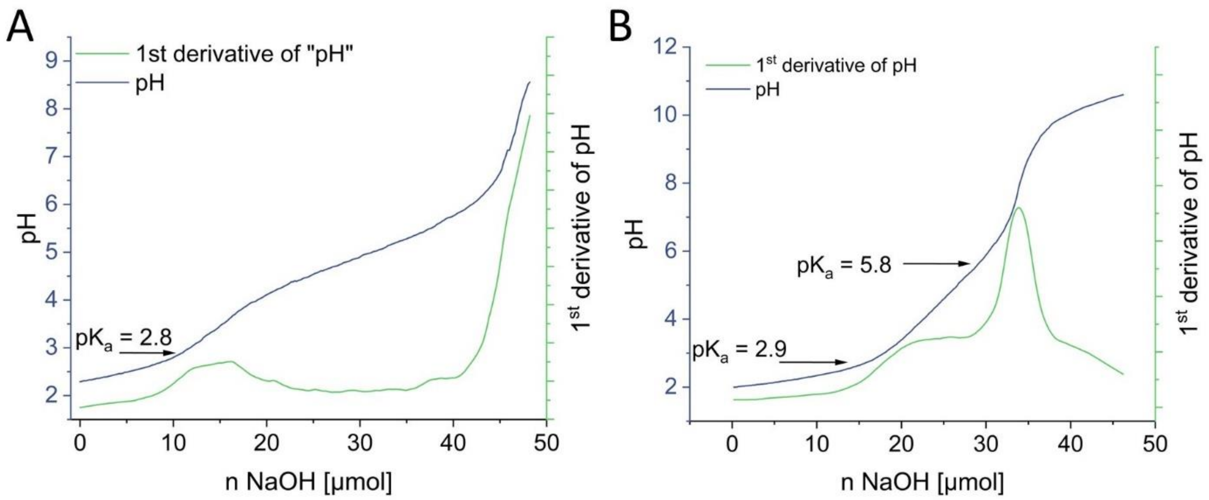

Figure S19. Titration curve and the first derivative of (A) P(Me3Lys-OH-AAm) and (B) P(Lys-OH-AAm) (5 mg mL^{-1} in H_2O) with NaOH (0.1 M in H_2O).

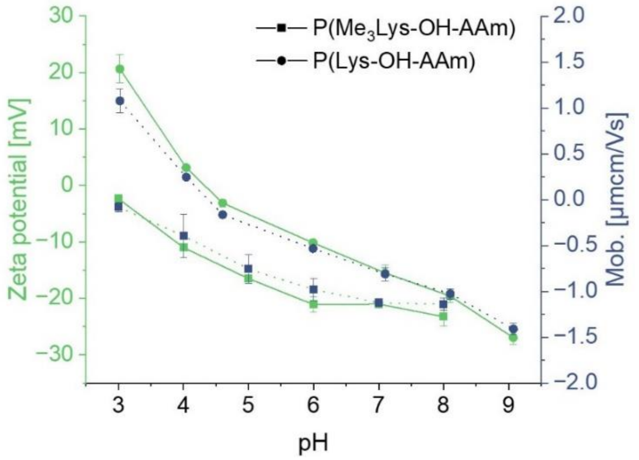

Figure S20. The ζ-potential (green) and electrophoretic mobility (blue) of 2 mg mL^{-1} aqueous solutions of P(Me_3Lys-OH-AAm) (squares) and P(Lys-OH-AAm) (circles) at indicated pH values.

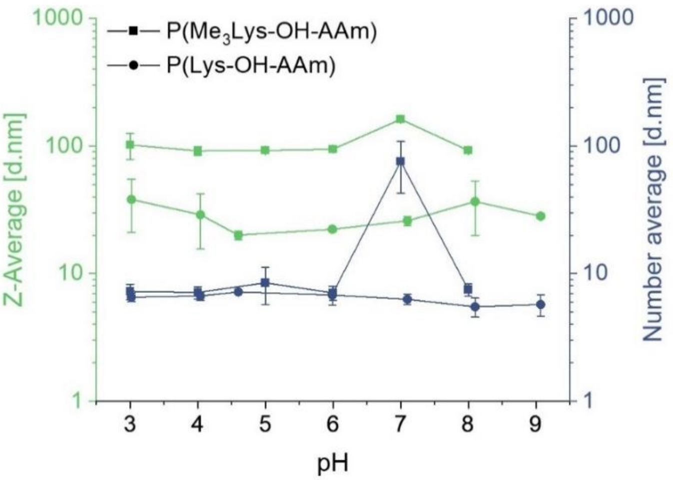

Figure S21. Intensity weighted (green) and number mean (blue) sizes of 2 mg mL^{-1} aqueous solutions of P(Me_3Lys-OH-AAm) and P(Lys-OH-AAm) at indicated pH values.

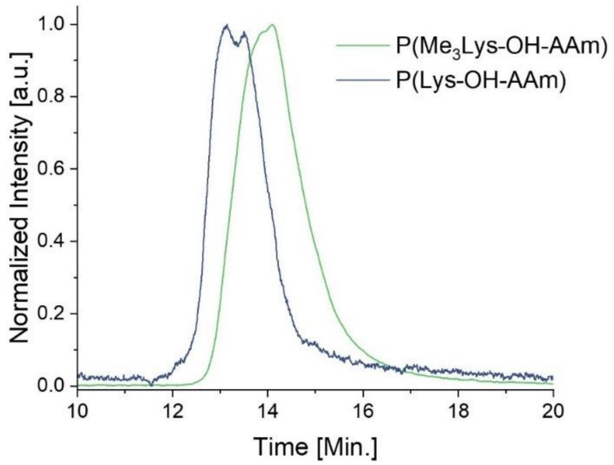

Figure 22. HPLC chromatograms (acetonitrile/water gradient, 0.1v% TFA). Spectra shows the normalized fluorescence intensity of eluting Cy5-labeled polymers (λ_{ex} = 640 nm; λ_{em} = 680 nm).

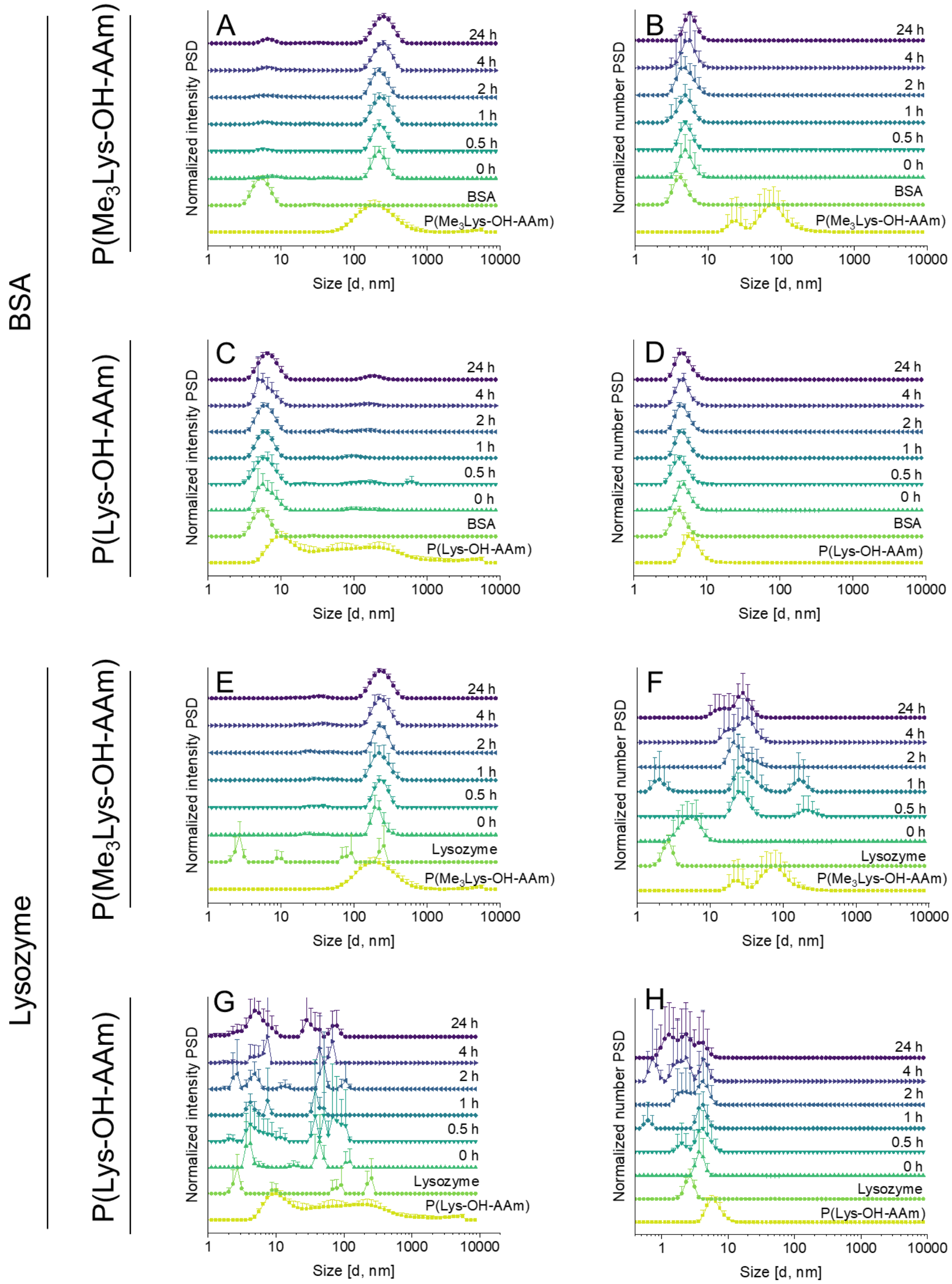

Figure S23. Intensity and number size distributions of polymer-protein-mixtures. The concentration of polymers and proteins was 0.5 mg mL^{-1} each in DPBS. Sizes were analyzed by DLS measurements at 37 °C. Values (scatter) represent the mean and SD of five measurements with three runs each. Dashed lines do not represent measured values. A and B: P(Me_3Lys-OH-AAm) and BSA. C and D: P(Lys-OH-AAm) and BSA. E and F: P(Me_3Lys -OH-AAm) and lysozyme. G and H: P(Lys-OH-AAm) and lysozyme.

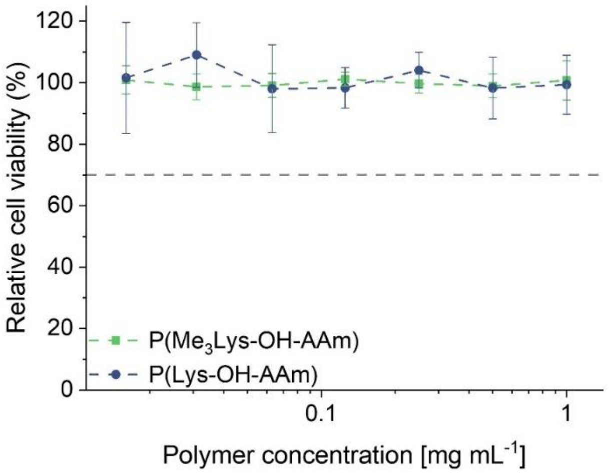

Figure S24. Cell viability of MDA-MB-231 breast cancer cells after incubation with P(Me_3Lys-OH-AAm) and P(Lys-OH-AAm), which were obtained *via* PPM, for 24 h. Cell viability was determined by MTT assay. Cells without polymer treatment served as negative control (NC, 100% cell viability). Values shown are relative to the NC. Cells treated with 20% DMSO served as positive control (PC, 0% cell viability, data not shown).
